# Impact of Nested Vark (Visual, Auditory, Read, Kinesthetic) Strategies on Parental Attachment, Anxiety, Maternal and Fetal Outcome Among First Time Childbearing Couples: A Randomized Controlled Trial

**DOI:** 10.7759/cureus.29257

**Published:** 2022-09-17

**Authors:** Dhanalakshmi V, Nalini S J, Anitha Rani M

**Affiliations:** 1 Obstetrics and Gynaecology, Apollo College of Nursing, The Tamilnadu Dr MGR Medical University, Chennai, IND; 2 Obstetrics and Gynaecology Nursing, Sri Ramachandra Institute of Higher Education and Research, Chennai, IND; 3 Community Medicine, Sri Ramachandra Institute of Higher Education and Research, Chennai, IND

**Keywords:** fetal outcomes, maternal outcomes, parental anxiety, parental attachment, kinesthetic strategies, read, auditory, visual

## Abstract

The transition to parenthood is not only a tremendous joy but also great stress to a mother (especially a primigravida) both physically and psychologically. The purpose of this research is to improve confidence in first-time childbearing couples, increase parental attachment, alleviate anxiety and promote the maternal and neonatal outcomes of delivery. In a randomized controlled trial design, simple randomization was utilized to enroll 300 first-time childbearing couples who have completed the 24^th^ week of gestation (150 in control and experimental arms). Nested refers to fully contained antenatal exercises, kick chart monitoring, and the use of a preggy imitator. VARK strategies indicate visual (information exhibited through demonstration), auditory (preference for information that is heard or spoken through lectures), read (information displayed as words, text-based input and output), and kinesthetic (preference for gathering information through experience and practice, simulated real, through concrete personal experiences) strategies along with routine care were executed on the experimental group. Data was collected using Condon’s Parental Attachment Scale and Perinatal Anxiety Scale during the pre-test at the 24^th^ week and post-tests at the 32^nd^, 33-34, and 36-38 weeks of gestation respectively, after the intervention through the self-administration method. Maternal and fetal outcomes were identified through medical records within 24 to 48 hours after delivery. The pre-test parental attachment mean score was significantly increased in post-test (Mothers: 61.44±3.27 to 77.89±32.44, fathers: 51.44±1.93 to 58.89±24.86) the for experimental group after the intervention compared to the control group (Pre-test score mother: 61.41±2.98 to 66.43±31.55, father: 51.24±3.95 to 49.30± 23.55) at p <0.001. The pre-test anxiety mean score was significantly reduced after intervention (mother: 44.60±3.92 to 16.57± 8.18, father: 22.55±3.41 to 6.28±4.47) for the experimental group compared to the control group (mother=46.22±5.12 to 25.03± 13.89, father=22.62±3.39 to 17.92± 9.92) at p <0. 001. The study findings recommend VARK strategies to be advocated with existing hands-on childbirth preparation classes for childbearing couples.

## Introduction

A pregnant woman undergoes not only physical changes, but also changes in mental health during pregnancy. The attachment between a pregnant mother and her developing fetus has been determined to be necessary because of the potential connection between antenatal attachment and parental behavior both, before and after birth [1]. The change of the motherhood role requires a dynamic organization of mental health to adapt and care for the newborn [2]. According to a report from Brazil, the anxiety prevalence rate was 26.8% of antenatal mothers, and 42.9 % of pregnant mothers were mostly diagnosed during the third trimester [3]. In Bangalore, the prevalence rate of pregnancy-related anxiety was identified in 195 antenatal mothers out of 380 mothers [4]. New fathers reported that they had difficulties in the balance of competing demands of family, work, and meeting their own needs. Apart from this, they struggled to maintain impaired relationships and felt detached from their partners following the delivery of their own baby [5,6]. Maternal anxiety during pregnancy leads to great adverse outcome during delivery in terms of preterm birth and low birth weight, which is evidenced in twelve studies totaling 17,304 antenatal mothers. The outcome of the study reported preterm birth data; concurrently 6 studies gave a result of Low-Birth-Weight data [7]. Every child on the provision of a conducive and enabling environment may blossom into an ever-fragrant flower to shine in all phases of life [8]. Hence the mother’s anxiety during the antenatal period has a direct impact on the child’s future behaviour.

At times, changes in mental health turn into pregnancy-specific anxiety (PSA). This was evidenced in a prospective study that explored the findings that the highest prevalence of PSA was found between the age group of 18-35 years, especially during the third trimester of pregnancy [9]. In the current study, the objectives of the study were:

Phase 1: design and develop a preggy imitator and validate its use among professionals and end users,

Phase 2: determine the impact of VARK strategies on parental attachment and anxiety among first-time childbearing couples,

Phase 3: evaluate the impact of VARK strategies on maternal and fetal outcomes among first-time mothers,

Phase 4: determine the relationship of parental attachment with anxiety among first-time childbearing couples in both, the control group and experimental group, and

Phase 5: associate selected background variables with the parental attachment and anxiety of first-time childbearing couples in both, the experimental and control groups.

We used VARK (visual, auditory, read, and kinesthetic) strategies which include antenatal exercises, kick-chart monitoring, and the use of a preggy imitator among first-time childbearing couples. There was a paucity of studies on this selected topic of the impact of nested VARK (Visual, Auditory, Read, Kinesthetic) strategies on parental attachment, anxiety, and maternal and fetal outcome among first-time childbearing couples, especially in Tamil Nadu.

## Materials and methods

To accomplish the objectives of the study, quantitative research with an evaluative approach was adopted. An approval to conduct the study was obtained from the Sri Ramachandra University’s Ethical Committee as well as Ethical Committee clearance was obtained from Apollo Hospitals, Chennai. The ethical consideration criteria were based on the Indian Council of Medical Research guidelines for biomedical research in human beings. Information essential for consent included a description of the purpose of the study, possible benefits to the participant, confidentiality, the participant’s right to withdraw from the study, and voluntary consent. The researcher introduced herself to the first-time childbearing couples and obtained informed consent from those who met the inclusion criteria.

Phase 1: development and validation of preggy imitator

The new intervention preggy imitator, one of the VARK techniques, was tested to see how well-liked it was by the partners of primigravida women. Before being utilised by partners of primigravida women, the preggy imitator was approved by seven experts. The researcher identified the mothers with their partners who met the inclusion criteria and selected 30 mothers in the setting for the study through the purposive sampling technique. After the initial introduction, the researcher obtained consent from the partners of the primigravida mothers to participate in the study. Assurance was given regarding confidentiality before the data collection procedure. The data was collected by using predetermined tools such as demographic variable proforma and obstetrical variable proforma. Following this, the preggy imitator garment was administered individually one by one to the partner of all the primigravida mothers for a duration of 10-15 min. The partners were assessed by the researcher during the sessions using the checklist. Then the level of satisfaction with the preggy imitator was assessed for the partner of the primigravida mother by a rating scale.

Phase 2: determine the impact of VARK strategies on parental attachment and anxiety among first-time childbearing couples

A randomized controlled trial design was adopted for conducting this study. The expectant fathers’ paternal attachment mean score was 80.62 with a standard deviation of 15.4, assuming that the increase in paternal attachment mean score was 86.62 with a standard deviation of 14 [10].

The sample size was calculated using the formula

Z = 2Sp2(z1 - α/2 + z1 - β)/μd2,

where Sp2 = S12 + S22/2,

S12 = standard deviation in the first group, S22 = standard deviation in the second group, μd2 = mean difference between the samples, α = significance level (95%), and (1 - β) = power.

The estimated sample size was approximately 126 subjects in each group. Expecting 20% will be lost to follow-up, the sample size was increased by 20%. The final sample size arrived at was 150 in each group. Simple randomization (coverslip method) was utilized to assign samples to respective groups. A planned enrolment of 300 participants, 150 per group, was randomly assigned to the control and experimental arms. The total sample was divided into 6 blocks, with each block consisting of 50 samples, 25 in the study and 25 in the control groups. As the mothers became samples, the investigator requested them to pick a chit from the box. As per the letters indicated in the selected chit, women were randomly assigned to the control and experimental groups. When the assignment of samples to the first block was completed, the second block was considered. After the selection of the first-time childbearing couples, they were given information on the Nested VARK strategies on parental attachment, anxiety, and maternal and fetal outcomes. VARK strategies sensitized the senses which created interest to learn and understanding among first-time childbearing couples. These strategies are tailor-made and given in a shorter version in Table 1.

**Table 1:** Activities of manipulation

Steps/weeks of gestation	Interventions	Modality	Strategy used
Step I (24th week)	Antenatal exercises	Video-assisted teaching cum demonstration	Visual, auditory, kinesthetic strategy
Step II (32ndweek)	Kick chart monitoring	Lecture cum demonstration	Visual, auditory, read, kinesthetic strategy
	Preggy imitator	Lecture, demonstration, and simulated practice	Visual, auditory, kinesthetic strategy
Step III (33–34 weeks)	Reinforcement of VARK strategies		

The researcher administered these three interventions (antenatal exercises, kick chart monitoring, and use of a preggy imitator) along with routine education to the experimental group. Routine antenatal education includes normal childbirth, the position of childbirth and labor, and relaxation and massage techniques. In relation to the first intervention schedule, antenatal exercises were taught by the researcher on a one-to-one basis to the first-time childbearing mother in the experimental group at 24 weeks of gestation with the help of video-assisted teaching lasting for 9 min, followed by a 30-min demonstration which was enacted by the first-time childbearing mother. The mothers were instructed to practice daily.

With regard to the second intervention schedule, kick chart monitoring and the use of a preggy imitator were taught by the researcher to the first-time childbearing couples at 32 weeks of gestation for 20-30 min. In kick chart monitoring intervention, the mothers were advised to choose one period during the day to count, especially when the baby is normally active, and instructed to count at the same time every day. This intervention was done for 15-20 min. Followed by kick chart monitoring intervention, preggy imitator intervention was administered to the partner of the first-time childbearing mother. He was advised to wear it and instructed to do 10 pre-planned activities for 10-15 min. The teaching strategies were reinforced during follow-up visit,s especially at 33-34 weeks of gestation for 10-15 min.

The control group's first-time parents were instructed to continue receiving routine antenatal care and to attend routine antenatal education, which covers topics like typical childbirth, the position of childbirth throughout labor, relaxation techniques, and massage.

Subjects who fulfilled the inclusion criteria during the period of study were considered as samples.

**Figure 1:**
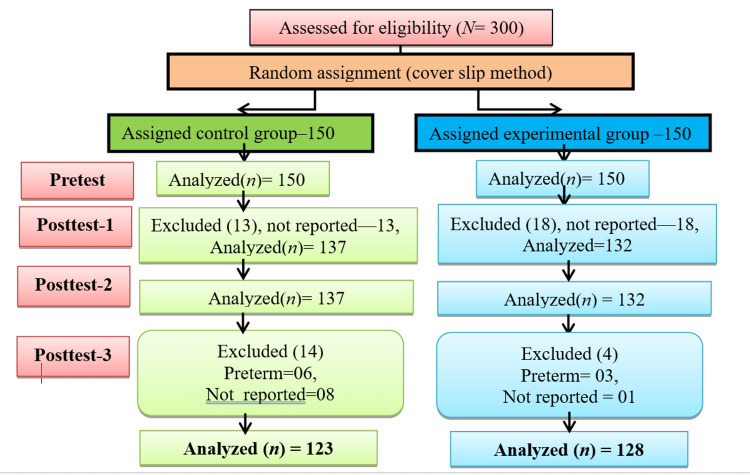
Flow chart for participant’s recruitment

They were randomly assigned to the study or control group. The researcher completed the assessment of anxiety and attachment for the control group at the same time intervals as that of the experimental group in the same setting. To assign samples to the control and experimental groups, simple randomization (the coverslip method) was used. In this study, the target population was first-time childbearing women at 24 weeks of gestation and their partners. The accessible population was first-time childbearing couples who were included from the 24th week of gestation and who attended the antenatal outpatient department of the hospital, partners of all first-time childbearing mothers, medical and nursing experts were included in Phase-I. All first-time childbearing women at 24 weeks of gestation and their partners who met the inclusion criteria were randomized to Phase II. This study excluded childbearing couples who were suffering from high-risk pregnancies such as diabetes and hypertension and a mother’s partners who had a history of alcoholism and smoking. The demographic and obstetric variables had open or closed questions that were answered by the first-time childbearing couple. The data were collected from the mother and her partner during her antenatal visit at the 24thweek of gestation on the selection of subjects as samples. Condon’s Maternal Antenatal Attachment Scale (19 items) and Paternal Antenatal Attachment Scale (16 items) [11] were used to identify parental attachment. Scoring was interpreted such that the higher the score, higher the attachment, and the lower the score, lower the attachment.

The Mother Perinatal Anxiety Screening Scale (Mother-PASS) and Father Perinatal Screening Scale (Father-PASS) [12] were used to identify the anxiety level of the parents. Scoring was interpreted such that the higher the score higher the anxiety, and the lower the score lower the anxiety. These rating scales were administered during pretest and posttests. Pretest was assessed during the 24th week and posttests were assessed (1-3) at 32, 33-34, and 36-38 weeks, respectively, after the intervention. First-time couples were asked to select their answer based on their experience over the past month. If the couple did not come for the next visit or did not continue the treatment in the same setting, they were considered dropout samples. The dropout sample was also noted down as a whole number for each group. Data from the mother's medical records, which was obtained within 24 to 48 hours following the mother's delivery, were gathered using the Maternal and Fetal Outcome Variable Proforma. The information was taken from the mother's postpartum medical record and includes the results of cardiotocography (CTG), fetal heart rate, length of the first stage of labor, length of the second stage of labor, length of the third stage of labor, maternal outcome during delivery, gestational age at labor, and birth weight of the baby.

All the instruments were reviewed for content validity by medical and nursing experts and they were pilot tested to assess their usability and ease of administration.

**Table 2:** Schematic representation of data collection procedure O ‒Study group,# ‒ Control group, X1‒ Antenatal exercises intervention, X2 ‒Kick chart monitoring intervention, and use of preggy imitator, X3 –Reinforcement teaching

S. No.	Activities	24 weeks of gestation	32 weeks of gestation	33–34 weeks of gestation	Within 24-48 hours after delivery	After delivery
1	Informed consent Background variables Pretest	O #				
2	Interventions	O*X_1_ #*	O*X_2_	O*X_3_	O*X_3_	
3	Posttest		O #	O #	O #	
4	Maternal and neonatal outcome from records				36–38 weeks of gestation	O #

## Results

Phase-I

The majority of partners of the primigravida mothers were highly satisfied (90%) and most of them moderately satisfied regarding the quality of the preggy imitator (90%). Few of them were moderately satisfied related to the clarity of the presentation (10%) and easy-to-follow instructions (10%).

Phase-II

Descriptive analysis of demographic variables of mothers showed that around 82 (55%) of the control group and 77 (51%) in the experimental group were in the age group of 20-29. According to a descriptive examination of fathers, the majority of them were between the ages of 30-39 years, with 131 (87%) in the experimental group and 124 (83%) in the control group.

With regard to the comparison of maternal antenatal attachment levels between control and experimental groups in pretest and posttest-3, 87 (59%) in the control group and 88 (59%) in the experimental group had a high antenatal attachment in the pretest. Forty-two (28%) in the control group and 40 (26%) in the experimental group had a moderate attachment. Nineteen (14%) in the control group and 21 (14%) in the experimental group had a low attachment. Two (1%) in the control group and one (1%) in the experimental group had a very high attachment. None of them had a very low attachment in both the control and experimental groups. No significant difference was found between groups on maternal antenatal attachment in the pretest. In the posttest, the majority of them, 65 (51%) in the control group and 128 (100%) in the experimental group had a very high attachment. Forty-five (37%) had a high attachment and 13 (11%) had a moderate attachment in the control group. No one expressed low attachment and very low attachment in both the control group and the experimental group. Significant differences between the groups were shown by a chi-square value of 71.78 with a p-value of 0.001 (Table 3).

**Table 3:** Comparison of maternal antenatal attachment level between control and experimental groups in pretest and posttest-3 (N = 300) aControl group n = 123, experimental group n = 128. ***p < 0.001.

Maternal antenatal attachment level	Control group (n = 150)		Experimental group (n = 150)		χ^2^Value p-value
	No.	%	No.	%	
Pretest					0.48 0.921 NS
Very high attachment	2	1	1	1	
High attachment	87	59	88	59	
Moderate attachment	42	28	40	26	
Low attachment	19	14	21	14	
Very low attachment	0	0	0	0	
Posttest-3^a^					71.78*** 0.000 S
Very high attachment	65	51	128	100	
High attachment	45	37	0	-	
Moderate attachment	13	11	0	-	
Low attachment	0	0	0	-	
Very low attachment	0	0	0	-	

With respect to the comparison of paternal antenatal attachment levels between control and experimental groups in pretest and posttest-3, 119 (80%) in the control group and 125 (90%) in the experimental group had a moderate attachment in the pretest. Twenty-nine (19%) in the control group and 25 (10%) in the experimental group had a high attachment. Two (1%) in the control group had a low attachment. No one had a low attachment in the experimental group and very low attachment in the control and experimental groups. In posttest-3, 97 (79%) in the control group and 109 (85%) in the experimental group had a high attachment. Twenty-six (21%) in the control group and 10 (8%) in the experimental group had a moderate attachment. Nine (7%) in the experimental group and none of them in the control group had a very high attachment. None of them had a low attachment and very low attachment in both the control and experimental groups. The groups had significant differences exhibited by a chi-square value of 7.7461 with p < 0.005 (Table 4).

**Table 4:** Comparison of paternal antenatal attachment level between control and experimental groups in pretest and posttest-3 (N = 300) S, significant; NS, nonsignificant. ,aControl group n = 123, experimental group n = 128. **p < 0.01.

Paternal antenatal attachment level	Control group (n = 150)		Experimental group (n = 150)		χ^2^Value p-value
	No.	%	No.	%	
Pretest					0.430 0.511 NS
Very high attachment	0	0	0	0	
High attachment	29	19	25	10	
Moderate attachment	119	80	125	90	
Low attachment	02	01	0	0	
Very low attachment	0	0	0	0	
Posttest-3^a^					7.7461** 0.005 S
Very high attachment	0	0	9	07	
High attachment	97	79	109	85	
Moderate attachment	26	21	10	08	
Low attachment	0	0	0	0	
Very low attachment	0	0	0	0	

Table 5 compares the maternal antenatal attachment between the control group and experimental group in pretest and posttests. In posttests 2 and 3, there was a significant difference between the groups at p 0.05 and p 0.01 respectively. In post-tests 1 and 2, there was no statistically significant difference between the groups.

**Table 5:** Comparison of Maternal attachment between the control group and experimental group in pretest and posttests (N = 300) aControl group n = 137; experimental group 132, bControl group n = 137, experimental group n = 132, c Control group n = 123, experimental group n = 128.*p < 0.05, **p < 0.01.

Group	Pretest	^a^ Post-test-1	^b ^Post-test-2	^c ^Post test-3
Control Group	61.41±2.98	65.3 ± 24.15	67.09±26.52	66.43±31.55
Experimental Group	61.44±3.27	67.11±27.26	74.22±30.18	77.89±32.44
Unpaired ‘t-test	0.074	0.323	2.172*	3.103**
p-value	0.941	0.747	0.031	0.002

Table 6 compares paternal antenatal attachment based upon gestation weeks between the control group and the experimental group in pretest and posttests. There was no statistical difference in the pretest for both control and experimental groups, whereas there were statistical differences at p < 0.05 in posttest-1 and p < 0.01 in posttests-2 and -3 between control and experimental groups.

**Table 6:** Comparison of Paternal Attachment Mean score between the Control group and Experimental Group in Pretest and Posttests (N = 300) aControl group n = 137, experimental group n = 132,  bControl group n = 137, experimental group n = 132, cControl group n = 123, experimental group n = 128.*p < 0.05, **p < 0.01.

Group	Pretest	^a ^Post test-1	^b^ Post test-2	^C^ Post test-3
Control Group	51.24± 3.95	46.85±18.19	49.23±19.14	49.30±23.55
Experimental	51.44±1.93	52.51±21.61	55.70±22.93	58.89±24.86
Unpaired ‘t’ test &	0.558	2.451*	2.652**	3.429**
p value	0.577	0.015	0.008	0.001

With respect to the comparison of maternal anxiety levels between control and experimental groups in pretest and posttest-3, 129 (86%) in the control group and 115 (51%) in the experimental group had severe symptoms in the pre-test. In posttest-3, 10 (8%) in the control group and none in the experimental group had severe symptoms. With regard to mild-moderate symptoms and asymptomatic in posttest-3, the control group had 109 (89%) and 4 (3%) whereas the experimental group had 46 (36%) and 86 (64%), respectively. In the posttest-3, the groups' significant differences were shown by chi-square values of 106.33 at p < 0.001 (Table 7).

**Table 7:** Comparison of maternal anxiety level between control and experimental groups in pretest and posttest-3 (N = 300) aControl group n = 123, experimental group n = 128. ***p < 0.001.

Maternal anxiety	Control group (n = 150)		Experimental group (n = 150)		χ^2^ Value p-value
	No.	%	No.	%	
Pretest					4.3033 0.03803
Asymptomatic	0	0	0	0	
Mild-moderate symptoms	21	14	35	49	
Severe symptoms	129	86	115	51	
Posttest-3^a^					106.33*** 0.0001
Asymptomatic	4	3	82	64	
Mild-moderate symptoms	109	89	46	36	
Severe symptoms	10	8	0	0	

With reference to the comparison of paternal anxiety levels between control and experimental groups in pretest and posttest-3, 131 (87%) in the control group and 139 (92%) in the experimental group had mild-moderate symptoms in the pretest. Few of them, 15 (10%) in the control group and 10 (7%) in the experimental group, were asymptomatic. Very few of them, four (3%) in the control group and one (1%) in the experimental group, had severe symptoms. In posttest-3, 75 (61%) in the control group and 127 (99%) in the experimental group were asymptomatic, whereas 48 (39%) in the control group and 1 (1%) in the experimental group had mild-moderate symptoms. There was a significant difference between the groups (Table 8).

**Table 8:** Comparison of paternal anxiety level between control and experimental group in pretest and posttest-3 (N = 300) aControl group n = 123, experimental group n = 128.***p < 0.001.

Paternal anxiety	Control group (n = 150)		Experimental group (n = 150)		χ^2^ p-value
	No.	%	No.	%	
Pretest					3.037 0.2190
Asymptomatic	15	10	10	07	
Mild-moderate symptoms	131	87	139	92	
Severe symptoms	4	3	1	1	
Posttest-3^a^					59.842*** 0.000
Asymptomatic	75	61	127	99	
Mild-moderate symptoms	48	39	1	1	
Severe symptoms	0	0	0	0	

Table 9 shows that there was no statistical difference in Maternal Anxiety in the pretest for both control and experimental groups, whereas there was a statistical difference between the groups in posttest-1 at p < 0.01 and between the groups in posttests-2 and -3 at p < 0.001.

**Table 9:** Comparison of Maternal Anxiety Mean score between the Control group and Experimental Group in Pretest and Posttests (N = 300) aControl group n = 137, experimental group n = 132, bControl group n = 137, experimental group n = 132, cControl group n = 123, experimental group n = 128. ***p < 0.001.

Group	Pretest	^a^ Post test-1	^b ^Post test-2	^C^ Post test-3
Control Group	46.22±5.12	36.08±14.57	26.12±11.05	25.03±13.89
Experimental	44.60±3.92	30.99±12.78	18.25±7.98	16.57±8.18
Unpaired ‘t’ test	3.072**	3.218**	7.051***	6.436***
p-value	0.002	0.001	0.000	0.000

Table 10 shows that there was no statistical difference in Paternal Anxiety between the groups in the pre-test whereas there was a statistical difference between the groups in posttests-1-3 at p < 0.001.

**Table 10:** Comparison of Paternal Anxiety Mean score between the Control group and Experimental Group in Pretest and Posttests (N = 300) aControl group n = 137, experimental group n = 132, bControl group n = 137, experimental group n = 132, cControl group n = 123, experimental group n = 128. **p < 0.01.

Group	Pretest	Post test-1	Post test-2	Post test-3
Control Group	22.62±3.39	21.01±9.30	18.91±8.36	17.92±9.92
Experimental	22.55±3.41	15.38±3.40	9.53±2.28	6.28±4.47
Unpaired ‘t-test	0.170	6.954**	13.252**	13.107**
p-value	0.865	0.001	0.001	0.001

Table 11 shows that there was a significant difference in the components of maternal and fetal outcomes-Cardiotocography; FHR; durations of the first stage of labor, the second stage of labor, and third stage of labor. There was a statistical difference in maternal outcome during labor between the groups at p < 0.001 and gestation age at delivery at p < 0.05.

**Table 11:** Frequency and Percentage Distribution of Maternal and Fetal Outcomes Between Control and Experimental Groups S- significant, NS-Non-Significant. ***p < 0.001.

Maternal and fetal outcomes	Control group (n = 123)	Experimental group (n = 128)	χ2 Value df	p-value
	n(%)	n(%)		
Cardiotocography				
Normal	110 (89)	128(100)	-	-
Abnormal	13(11)	0		
FHR				
< 120	2(2)	0	-	-
120–160	121(98)	128(100)		
Duration of first stage of labor				
10–11 hr	5(6)	6(8)	0.323 1	0.5698
11–13 hr	82(94)	69(92)		
Duration of second stage of labor				
<1 hr	44(51)	62(83)	18.33*** 1	0.0000
>1 hr	43(49)	13(17)		
Duration of third stage of labor				0.0000
<20 min	55(61)	70(93)	20.72*** 1	
>20 min	32(37)	5(7)		
Maternal outcome during labor^a^				0.000
Normal vaginal delivery	40(29)	57(43)		
Assisted vaginal delivery	47(34)	18(14)	22.18*** 5	
Elective LSCS	36(26)	53(40)		
Emergency LSCS	8(6)	1(1)		
Preterm delivery	6(4)	3(2)		
Gestation age at delivery			16.46*** 1	0.000
36–38 weeks	121(98)	107(81)		
38–40 weeks	2(2)	21(19)		
Birth weight of the baby				0.1476 NS
<2.5 kg	17(14)	12	3.825 2	
2.5–3.5 kg	64(52)	82		
>3.5 kg	42(34)	34		

Table 12 reveals the correlation between maternal antenatal attachment and maternal perinatal anxiety for the control group and the experimental group in posttest-3. There was a strong negative correlation, which means that high maternal antenatal attachment scores go with low perinatal anxiety scores of mothers for both control and experimental groups.

**Table 12:** Correlation between maternal antenatal attachment and maternal anxiety for control group and experimental group in posttest-3

Variables (Control group)	Maternal anxiety (Posttest-3)	Variables (Experimental group)	Maternal anxiety (Posttest-3)
	r-value		r-value
Maternal antenatal attachment (posttest-3)	0.8373	Maternal antenatal attachment (posttest-3)	0.7483
p-value	0.00001	p-value	0.00001

Table 13 depicts the correlation between paternal antenatal attachment and paternal anxiety for control group and experimental group in posttest-3. There was a strong positive correlation, which means that high paternal antenatal attachment scores go with high perinatal anxiety scores of fathers for control group whereas there was a strong negative correlation, which means there was a tendency for high paternal antenatal attachment scores to go with low perinatal anxiety scores of fathers for experimental group. 

**Table 13:** Correlation between paternal antenatal attachment and paternal anxiety for the control group and the experimental group in posttest-3

Variables (Control group)	Paternal anxiety (Posttest-3)	Variables (Experimental group)	Paternal anxiety (Posttest-3)
	r-value		r-value
Paternal antenatal attachment (posttest-3)	0.8295	Paternal antenatal attachment (posttest-3)	-0.53
p-value	0.00001	p-value	0.00001

Association between selected background variables with parental attachment, anxiety, and maternal and fetal outcomes

Maternal antenatal attachment and BMI, paternal antenatal attachment and BMI, maternity anxiety and BMI, paternal anxiety and mothers' educational status, and paternal anxiety and fathers' age at marriage were all significantly correlated with one another in the experimental group (p=0.001).

Table 14 indicates that there was a significant association between maternal antenatal attachment & maternal anxiety with BMI in the experimental group at p < 0.01.

**Table 14:** Association of selected obstetric variables with posttest-3 maternal antenatal attachment and maternal anxiety in the experimental group (n = 128) S, significant; NS, non-significant.    **p < 0.01, ***p < 0.001. **p < 0.01.

Obstetric variables	Maternal antenatal attachment			f-value	Maternal anxiety			f-value
	No.	Mean	SD	p-value	No.	Mean	SD	p-value
Body mass index								
								<20	20	82.45	28.231	8.390***	20	18.35	8.622	4.658**
20–24	92	81.57	28.164	92	17.04	7.27	0.004					
25–29	16	52.57	47.25	16	12.5	11.447	S					
Antenatal visits								
				0.027				0.312
Up to four visits	12	76.42	35.72	0.87	12	17.83	8.695	0.578
More than four visits	116	78.02	32.286	NS	116	16.46	8.153	NS

Table 15 indicates that there was a significant association of Paternal antenatal attachment and Paternal anxiety with BMI in the experimental group at p < 0.01. 

**Table 15:** Association of selected obstetric variables with posttest-3 Paternal antenatal attachment and Paternal anxiety in the experimental group (n = 128) S, significant; NS, non-significant.    **p < 0.01, ***p < 0.001. **p < 0.01.

Obstetric variables	Paternal antenatal attachment			f-value	Paternal anxiety			f-value
	No.	Mean	SD	p-value	No.	Mean	SD	p-value
Body mass index								
<20	20	61.3	21.384	8.373***	20	8.6	6.134	3.459*
20–24	92	61.91	21.781	0	92	6.06	3.69	0.018
25–29	16	39.21	35.354	S	16	5.64	6.368	S
Antenatal visits								
				0.016				1.757
Up to four visits	12	59.75	28.111	0.901	12	7.92	6.762	0.187
More than four visits	116	58.81	24.666	NS	116	6.14	4.221	NS

Table 16 exhibits that there was a significant association of paternal anxiety with marriage age in years of fathers in the experimental group at p < 0.05 level. 

**Table 16:** Association of selected demographic variables of fathers with posttest-3 paternal anxiety in the experimental group (n = 128) S, significant; NS, non-significant.  *p < 0.05.

Demographic variables	Paternal anxiety			f-value
	No.	Mean	SD	p-value
Age in years				
				0.648
20–29	11	7.05	3.88	0.422
30–39	117	6.17	4.553	NS
Marriage age in years				
				21–25	1	9		2.672*
25–30	55	7.17	5.028	0.072
>30	72	5.54	3.874	S
Occupation				
Govt employed	8	6.3	0.949	0.058
Private employed	91	6.2	4.598	0.943
Self-employed	29	6.49	4.74	NS
Nature of work				
				Shift duty	10	5.46	4.719	0.278
Night duty	23	6.57	5.049	0.758
Day duty	96	6.3	4.301	NS
Source of information				
				1.181
Relatives	73	5.93	4.106	0.279
Friends	55	6.73	4.891	NS

## Discussion

Phase-1

The research findings of the preggy imitator have the evidence to use and rationalize the practice for the main study. The present findings are consistent with the findings of the study in which Ford Motor Company asked its engineers (especially males) to wear the empathy belly, a simulator that helps them to recognize the symptoms of pregnancy, i.e. hands-on training- backache, pressure in the bladder area, the 30 or so pounds of extra weight and feel movements which mimic like fetal movements and kicking [13]. Yet another study done in India reported that during antenatal teaching programmes, partner involvement, especially, helps to meet the health needs of both the mother and child. Education about the competencies of the fetus increases the support and attachment to the unborn child. The parenting role increases the bond, reinforces the idea, and engages the partner to give more support during delivery [14].

Phase-2

The post-test of maternal antenatal attachment showed that the majority of mothers, 65 (51%) in the control group and 128 (100%) in the experimental group had a very high attachment. In relation to paternal antenatal attachment (posttest-3), 97 (79%) in the control group and 109 (85%) in the experimental group had a high attachment. With respect to the comparison of maternal anxiety levels between control and experimental groups, 129 (86%) in the control group and 115 (51%) in the experimental group had severe symptoms in the pre-test. In posttest-3, 10 (8%) in the control group and none in the experimental group had severe symptoms. With reference to the comparison of paternal anxiety levels between control and experimental groups, 131 (87%) in the control group and 139 (92%) in the experimental group had mild-moderate symptoms in the pre-test. The current study findings align with the findings of the relationship between social support and symptoms of mental illness (trait anxiety, maternal distress) and antenatal fetal attachment. The findings of the study mentioned above suggested that a variety of interventions could increase maternal-fetal bonding by lowering anxiety and enhancing social support [15].

Most women (99%) in the third trimester had a moderate to severe degree of PSA as evidenced by the highest mean scores of PSA [16]. The fetal movement counting in the third trimester was probed and the end result was a greater reduction of anxiety during the third trimester i.e between 28-37 weeks of gestation among first-time child-bearing mothers [16,17]. This result was supported by an evaluative study on the impact of expectant fathers’ training on paternal-fetal attachment (PFA). The training of fathers in attachment skills leads to increased paternal fetal attachment and a lower anxiety score [18]. Anxiety disorder and depression during pregnancy were not strong risk factors for adverse neonatal outcomes, although a low Apgar score in offspring of women with anxiety disorder may indicate poor neonatal adaptation [19].

This study brings out the positive result of VARK Strategies (Visual, Auditory, Read, Kinesthetic strategy) during pregnancy to alleviate anxiety and develop more bonding to an unborn child for better Maternal and Neonatal Outcomes of CTG, FHR, durations of the first stage of labor, the second stage of labor, and third stage of labor. There was a statistical difference in maternal outcome during labor between the groups at p < 0.001 and gestation age at delivery at p < 0.05.

Limitations of the study

We found it difficult to conduct interviews with both husband and wife in subsequent visits during follow-up. Future studies are needed to further confirm and generalize these results.

## Conclusions

VARK strategies assisted in strengthening parental fetal attachment and reducing parental anxiety in addition to providing routine care for antenatal mothers. In addition, this effective strategy was administered repeatedly in multiple interventions from the 24th week of gestation till the time of delivery. The multiple strategies used in this study appear to be more effective than a single strategy to improve parental fetal attachment and allay parental anxiety over an unborn child. Our study findings confirmed the hypothesized outcome that maternal and paternal antenatal attachment significantly increased after the administration of the VARK strategy. In addition, maternal and paternal anxiety significantly reduced after the implementation of the VARK strategy. The results of this study are clinically significant for parental attachment and anxiety, which aid in creating training and treatment programs. Due to several factors and interventions, we chose not to examine the biochemical variables of this study, such as epinephrine, norepinephrine, cortisol, and cholesterol. 

## References

[1] Condon JT, Corkindale C (1997). The correlates of antenatal attachment in pregnant women. Br J Med Psychol.

[2] Stern DN (2020). The motherhood constellation: A unified view of parent-infant psychotherapy.

[3] Silva MM, Nogueira DA, Clapis MJ, Leite EP (2017). Anxiety in pregnancy: prevalence and associated factors. Rev Esc Enferm USP.

[4] Nath A, Venkatesh S, Balan S, Metgud CS, Krishna M, Murthy GVS (2019). The prevalence and determinants of pregnancy-related anxiety amongst pregnant women at less than 24 weeks of pregnancy in Bangalore, Southern India. Int J Women's Health.

[5] Setodeh S, Pourahmad S, Akbarzadeh M (2017). A study of the efficacy of fathers’ attachment training on paternal-fetal attachment and parental anxiety. Fam Med Prim Care Rev.

[6] Baldwin S, Bick D (2018). Mental health of first time fathers - it's time to put evidence into practice. JBI Database System Rev Implement Rep.

[7] Ding XX, Wu YL, Xu SJ (2014). Maternal anxiety during pregnancy and adverse birth outcomes: a systematic review and meta-analysis of prospective cohort studies. J Affect Disord.

[8] Vijayalakshmi K, Muniappan K. (2016). Parental involvement and achievement of secondary school students. Int J Indian Psychol.

[9] Madhavanprabhakaran GK, D’Souza MS, Nairy KS. (2015). Prevalence of pregnancy anxiety and associated factors. International Journal of Africa Nursing Sciences.

[10] Kaur S, Sagar MN (2017). Comparative study to assess the maternal and paternal fetal attachment among the expectant mothers and fathers. Int J Reprod Contrcept Obstet Gynecol.

[11] Condon JT (1993). The assessment of antenatal emotional attachment: development of a questionnaire instrument. Br J Med Psychol.

[12] Somerville S, Byrne SL, Dedman K (2015). Detecting the severity of perinatal anxiety with the Perinatal Anxiety Screening Scale (PASS). J Affect Disord.

[13] Waytz A. (2016). The limits of empathy. Harvard Business Review.

[14] Toosi M, Akbarzadeh M, Sharif F (2014). The reduction of anxiety and improved maternal attachment to fetuses and neonates by relaxation training in primigravida women. Women’s health bulletin.

[15] O’Leary J. (2020). Fathers/partners: Psychological adjustment during pregnancy. https://infantcrier.mi-aimh.org/fatherspartners-psychological-adjustment-during-pregnancy/.

[16] Delaram M, Shams S (2016). The effect of foetal movement counting on maternal anxiety: A randomised, controlled trial. J Obstet Gynaecol.

[17] Nosraty A, Mirzakhani K, Golmakani N (2019). Effect of attachment training on paternal-fetal attachment. Journal.

[18] Akbarzade M, Setodeh S, Sharif F, Zare N (2014). The effect of fathers’ training regarding attachment skills on maternal-fetal attachments among primigravida women: a randomized controlled trial. Int J Community Based Nurs Midwifery.

[19] Berle JØ, Mykletun A, Daltveit AK, Rasmussen S, Holsten F, Dahl AA (2005). Neonatal outcomes in offspring of women with anxiety and depression during pregnancy. A linkage study from The Nord-Trøndelag Health Study (HUNT) and Medical Birth Registry of Norway. Arch Womens Ment Health.

